# Histamine and Serotonin Levels in Bone Marrow Stem Cells Niche as Potential Biomarkers of Systemic Mastocytosis and Myeloproliferative Disorders

**DOI:** 10.1007/s12015-022-10502-0

**Published:** 2022-12-29

**Authors:** Tomasz Janus, Urszula Korbal, Maciej Żukowski, Agnieszka Lewosiuk, Katarzyna Koper, Agnieszka Żukowska, Katarzyna Brzeźniakiewicz-Janus

**Affiliations:** 1 Department of Forensic and Clinical Toxicology, Pomeranian Medical University in Szczecin, Al. Powstańców Wlkp. 72, 70-111 Szczecin, Poland; 2 Department of Anesthesiology, Intensive Therapy and Poisoning, Pomeranian Medical University in Szczecin, Szczecin, Poland; 3 Department and Clinic of Hematology, Oncology and Radiotherapy of theUniversity of Zielona Góra, Zielona Góra, Poland; 4 Department of Infection Control, District General Hospital in Stargard, Stargard, Poland

**Keywords:** Histamine, Serotonin, Biomarker, Mastocytosis, Bone marrow

## Abstract

Bone marrow studies currently provide a lot of valuable information in the diagnostics of hematological diseases including hematopoietic stem cells disorders. Our studies on low-molecular weight organic compounds in bone marrow stem cell niche in various pathogenic conditions, revealed relatively high variability of histamine levels in different groups of hematological diseases. It was also found that serotonin levels were significantly lower than those typically measured in peripheral blood as well as many have the influence on stem cells proliferative potential. This paper presents findings from quantitative and statistical analyses of histamine and serotonin levels. Bone marrow collected from patients undergoing routine diagnostic procedures for hematological diseases and receiving inpatient treatment were analyzed. Histamine and serotonin levels were measured using hydrophilic interaction liquid chromatography (HILIC) coupled with tandem mass spectrometry. Obtained data were analyzed statistically and correlated with the diagnosed groups of hematological diseases and the parameters of complete blood counts. Histamine was found in all tested samples, including those from patients without malignancy, and the reported levels were comparable to the reference values in blood. This observation allows us to assume that bone marrow cells can produce and accumulate histamine. Moreover, the statistical analysis revealed a significant relationship between histamine levels and diagnosed mastocytosis, and between histamine levels and myeloproliferative neoplasms. Different results were obtained for serotonin, and its concentrations in most cases were below the limit of quantification of the method used (< 0.2 ng/mL), which can only be compared to peripheral blood plasma. In a few cases, significantly higher serotonin levels were observed and it concerned diseases associated with an increased number of megakaryocytes in the bone marrow.

## Introduction

Bone marrow tests are fundamental for the diagnosis of many hematological diseases. Bone marrow is also a valuable material when searching for new biomarkers facilitating the correct classification of diseases including hematopoietic stem cells disorders. According to the Biomarker Definition Working Group, a biomarker is a characteristic that is objectively measured and evaluated as an indicator of normal biological processes, pathogenic processes, or pharmacological response to a therapeutic intervention [1]. An important aspect in the development of a potential biomarker is the availability of the analytical material and low invasiveness of the sampling procedure.

Despite the fact that sampling bone marrow is a relatively invasive procedure, it is also a routine test used for more detailed hematological diagnostics, and its further use for the determination of other molecular parameters does not create any additional discomfort to the patient. While genetic markers are also commonly used in routine diagnostics, analyses of specific low-molecular weight compounds are less frequently practiced. Targeted metabolomics is becoming gold standard of stem cells and stem cells niche biology research. When conducting our targeted profiling studies of bone marrow samples for the presence of low-molecular weight compounds that could be used as potential biomarkers, we noticed significant differences in histamine levels between individual samples. Although histamine levels were measured in other materials such as plasma, blood, urine, gastric juice or cerebrospinal fluid [2–6], for the first time we show here the targeted metabolomic determination of histamine in bone marrow hematopoietic stem cells niche.

Histamine is a biogenic amine synthesized from L-histidine, and its biosynthesis takes place mainly in mast cells and basophils, where it is also accumulated [7]. On the other hand, serotonin is associated mainly with its activity in the nervous system, and less frequently with its physiological cellular function and its transport and accumulation by thrombocytes. It appears that histamine and serotonin levels measured in bone marrow niche, where storage cells for both of these compounds are produced, may be associated either with the cause of clinical abnormalities or an effect of the underlying pathogenic process. It should be noted that both histamine and serotonin levels in peripheral blood are relatively high, 20–200 ng/mL, which indicates their significant role in biological processes. On the other hand, routine laboratory tests focus on the analysis of morphotic blood parameters, and the presence of a specific group of cells (including bone marrow stem cells) in a certain number is considered normal, without the analysis of their functionality, which may result from an excess or deficiency of chemical cell components typical for them. A seemingly perfect example is systemic mastocytosis, which is known to be associated with the increased accumulation of mast cells in bone marrow and other organs, and these cells are characterized by a high content of histamine. An increased count of mast cells should therefore be associated with an increased concentration of histamine. Moreover, the targeted metabolic analysis of histamine and serotonin in bone marrow stem cell niche material might be the routine procedure in the most of the laboratories. Measured concentrations may reflect the normal activity of thrombocytes and granulocytes, resulting from the presence of these compounds in cells or the emergence of mature mast cells in an atypical location (e.g. bone marrow niche). Currently, many laboratories use selective and sensitive analytical techniques employing chromatographic separation and mass spectrometry, so histamine and serotonin can be detected in practice without the need for non-selective immunoassays. Thus, the determination of histamine and serotonin levels appears to perfectly complement a detailed hematological diagnostic procedure, along with other general tests such as complete blood count and bone marrow smear.

The aim of the present study was to determine histamine and serotonin levels in bone marrow collected from patients during routine hematological diagnostic procedures and to determine reference values, not related to the neoplastic disease process. Differences in the concentrations of both compounds in specific groups of diseases were assessed, and an attempt was made to determine the suitability of histamine and serotonin as potential diagnostic biomarkers.

## Methods

An analytical method employing liquid chromatography and tandem mass spectrometry (LC–MS/MS) was developed and validated for the simultaneous determination of histamine and serotonin levels. The method was validated separately for plasma and morphotic blood elements and it was characterized by very good analytical parameters, including linearity determined by the regression coefficient r^2^: 0.99984 for histamine and 0.999997 for serotonin. The limit of quantification (LOQ) for both histamine and serotonin was 0.2 ng/mL.

### Sampling and Storage of Bone Marrow Niche Material

Bone marrow niche material was sampled from patients hospitalized at the Department of Hematology, Oncology and Radiotherapy of the University of Zielona Góra, Multispecialist Provincial Hospital in Gorzów Wielkopolski. Bone marrow samples were collected by aspiration biopsy into Monovette EDTA KE test tubes and stored at (-24 °C) until further analysis. A total of 100 bone marrow samples were collected.

### Determination of Hematological Blood Parameters

Basic parameters of complete blood were analyzed using the Sysmex XN 2000 hematology system and dedicated diagnostic reagents. The population of white blood cells was determined by manual and automatic blood smears and presented in percentage of total cell count. The tests were carried out in the hospital laboratory on the day of the patient’s admission to the department.

### Preparation of Bone Marrow Samples for the Analysis of Histamine and Serotonin Levels

Prior to analysis, samples were thawed and thoroughly mixed (Classic Advanced Vortex Mixer, Velp Scientifica, Italy). Then, 50 µL of each sample was treated with 10 µL of sodium metabisulfite solution, 950 µL of mobile phase B, 2.5 µL of deuterated histamine d4 (internal standard, IS), at a concentration of 1 µg/mL and 2.5 µL of a 1 µg/mL deuterated serotonin d4 solution, mixed and centrifuged (Centrifuge 5417R, Eppendorf, Germany) at RCF 24,104 × g for 10 min. Next, 500 µL of supernatant was taken for LC–MS/MS analysis. Deuterated histamine and deuterated serotonin solutions were prepared by diluting stock standard solutions in methanol (Serotonin d4 hydrochloride, Toronto Research Chemicals, Canada, and Histamine- α,α,β,β d4 dihydrochloride Sigma Aldrich, US).

### LC–MS/MS Analysis

**Fig. 1:**
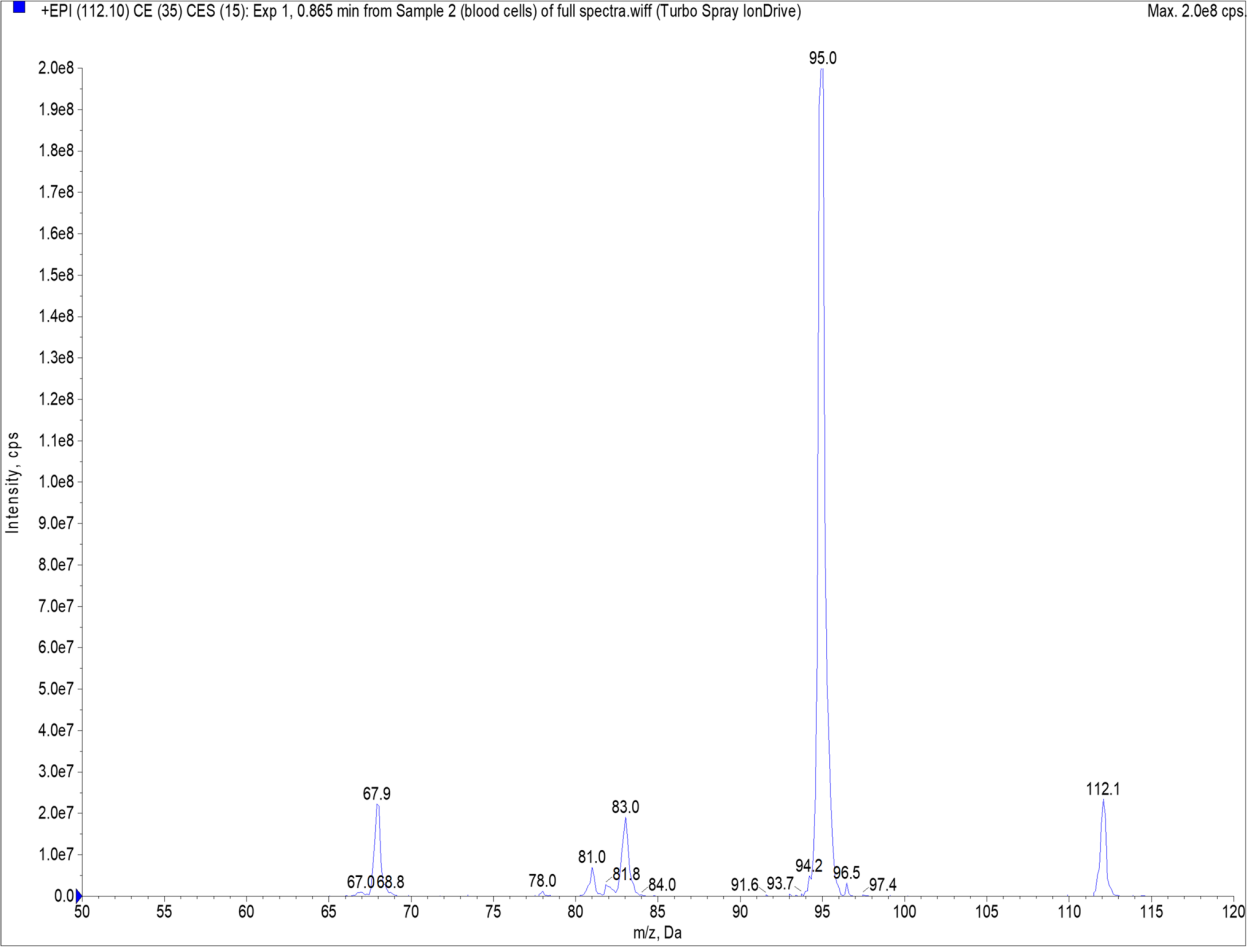
Histamine mass spectrum recorded in enhanced product ion mode for qualitative confirmation

**Fig. 2:**
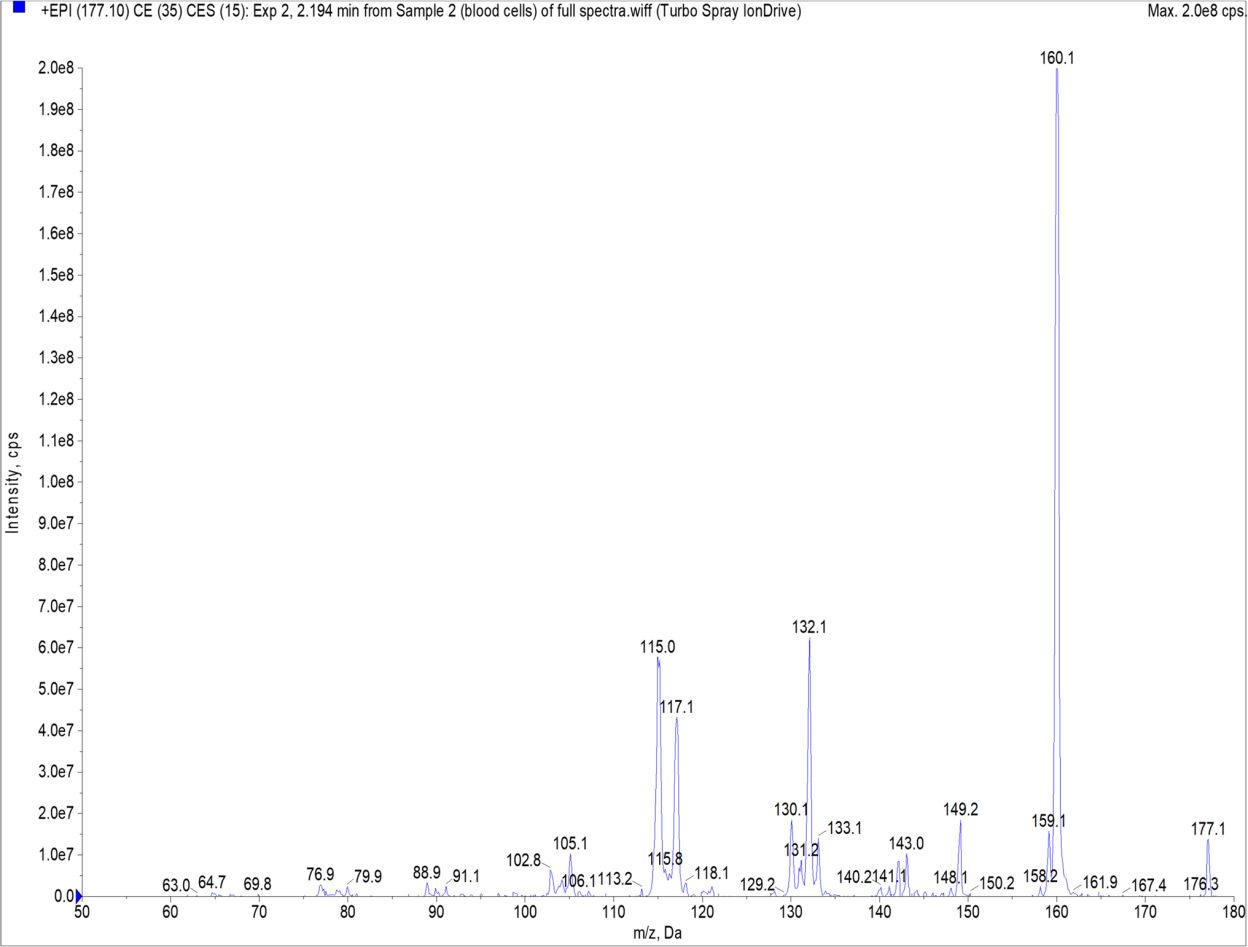
Serotonin mass spectrum recorded in enhanced product ion mode for qualitative confirmation

Analyses were performed using the ExionLC liquid chromatography system (AB Sciex, US) integrated with a QTRAP® 6500 + SelexIon tandem mass spectrometer (AB Sciex, US). Chromatographic separation was carried out on a SunShell HILIC-Amide column (2.6 µm, 2.1 i.d × 150 mm, ChromaNik Technologies, Japan). The chromatograph operating parameters were as follows: sample injection volume: 10 µL, equilibration time: 7 min, flow rate: 0.5 ml/min, oven temperature: 30 °C, mobile phase A: 80% water/10% ACN/10% ammonium formate buffer pH = 3.4 100 mM (v/v), mobile phase B: 90% acetonitrile (CAN)/10% buffer ammonium formate buffer pH = 3.4 100 mM (v/v). Gradient conditions: 0.00 min: 95.0% (B), 0.20 min: 95.0% (B), 1.40 min: 90.0% (B), 1.41 min: 75.0% (B), 3.30 min: 75.0% (B), 3.50 min: 30.0% (B), 6.30 min: 30.0% (B), 6.60 min: 95.0% (B), 7.00 min: 95.0% (B). The gradient resulted in the retention times of histamine 2.72 ± 0.01 min and serotonin 1.35 ± 0.00 min. The MS instrument operated in the positive mode, with the optimized settings for: ion source: ESI, curtain gas: 35 psi, ion spray voltage (IS): 4200 kV, heated gas temperature (TEM): 420 °C, ion source gas 1: 60 psi, ion source gas 2: 50 psi, entrance potential (EP): 10, collision cell exit potential (CXP): 10, dwell time: 50 ms. Declustering potential (DP) was 45 V for histamine-d4, histamine qualifier and quantifier, 5 V for serotonin-d4 and serotonin quantifier, and 120 V for serotonin qualifier. Collision Energy (CE) was 20 V for histamine-d4 and histamine quantifier, 30 V for histamine qualifier, 14 V for serotonin-d4, 13 V for serotonin quantifier, and 30 V for serotonin qualifier. Data acquisition was achieved in multiple reaction monitoring scanning mode (MRM). The mass transitions were m/z 116.100/99.100 for histamine-d4, m/z 112.100/95.100 as the quantifier for histamine, m/z 112.100/68.000 as the qualifier for histamine, m/z 181.100/164.100 for serotonin-d4, m/z 177.100/160.100 as the quantifier for serotonin, and m/z 160.100/115.100 as the qualifier for serotonin. In addition, both analytes were qualitatively confirmed by correlation of full enhanced product ion spectra measured for histamine (Fig. 1) and serotonin (Fig. 2) with database. Analyst Software provided by the manufacturer (SCIEX OS 1.7.0) was used for data analysis.

### Statistical Analysis

The statistical analysis was performed with R 4.1.2 software [8]. For statistical purposes, patients were grouped according to their diagnosis: myeloproliferative neoplasms, myelodysplastic syndromes, acute leukemias, lymphoproliferative diseases, other non-malignant hematological diseases, and mastocytosis. Correlations between quantitative variables were assessed using the Spearman correlation coefficient, and the values of quantitative variables in 6 groups were compared using the Kruskal–Wallis test. The significance of differences between groups was verified using the post-hoc Dunn's test. Differences were considered significant at p value < 0.05.

## Results

### Histamine and Serotonin Concentrations in Marrow Bone

Concentrations measured in all samples were 344.11 ± 959.09 ng/mL for histamine and 0.61 ± 3.69 ng/mL for serotonin (mean ± standard deviation). Histamine levels in 67% of all the samples were within the range of 20–200 ng/ml (blood normal levels), whereas 83% of all serotonin measurement and 97% for non-malignant hematological diseases, were below detection limit (0.2 ng/mL).

### Correlation Between Histamine, Serotonin Concentration and Hematological Disease Groups

**Fig. 3:**
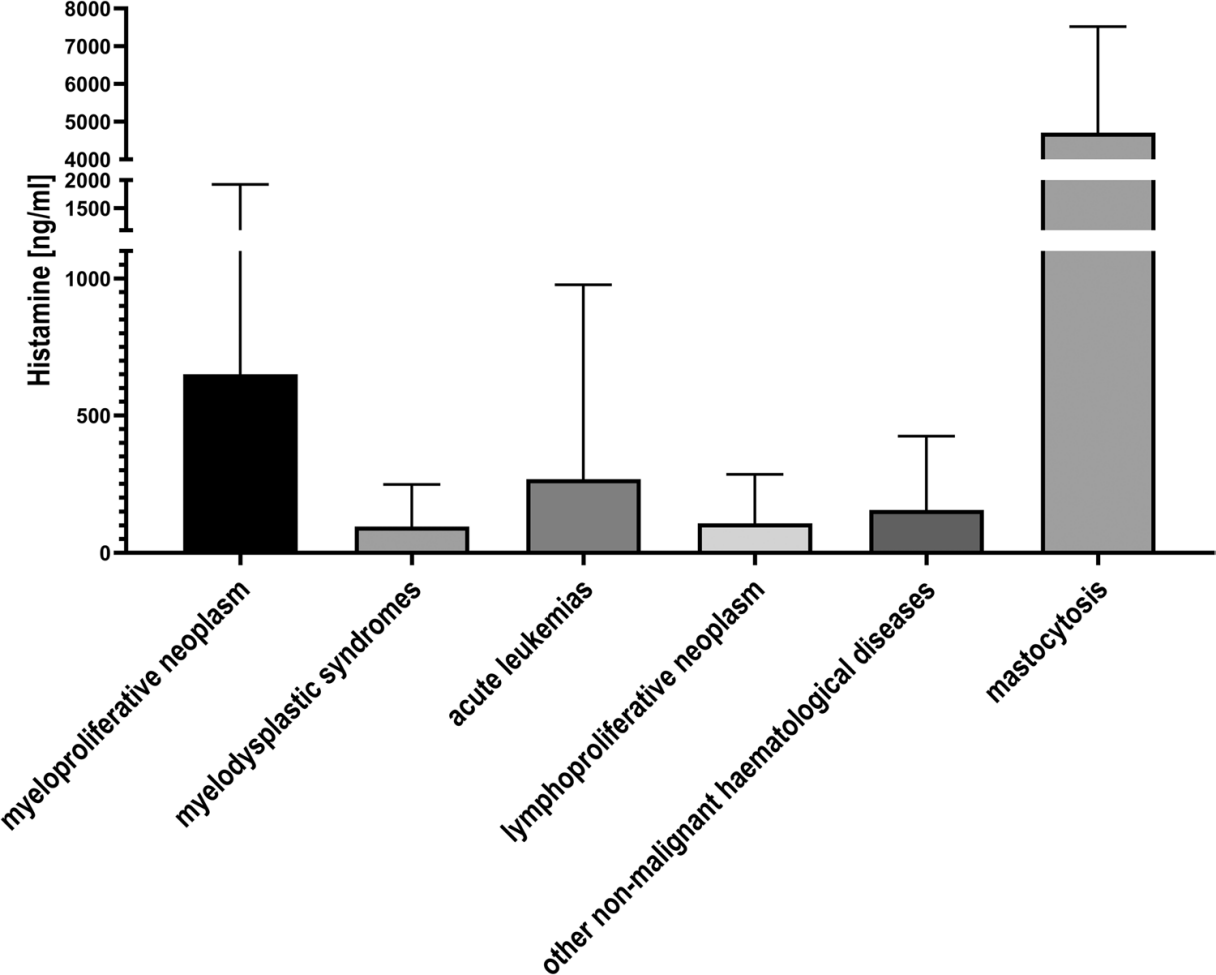
Box plot representing the correlation between histamine levels and groups of hematological diseases

**Fig. 4:**
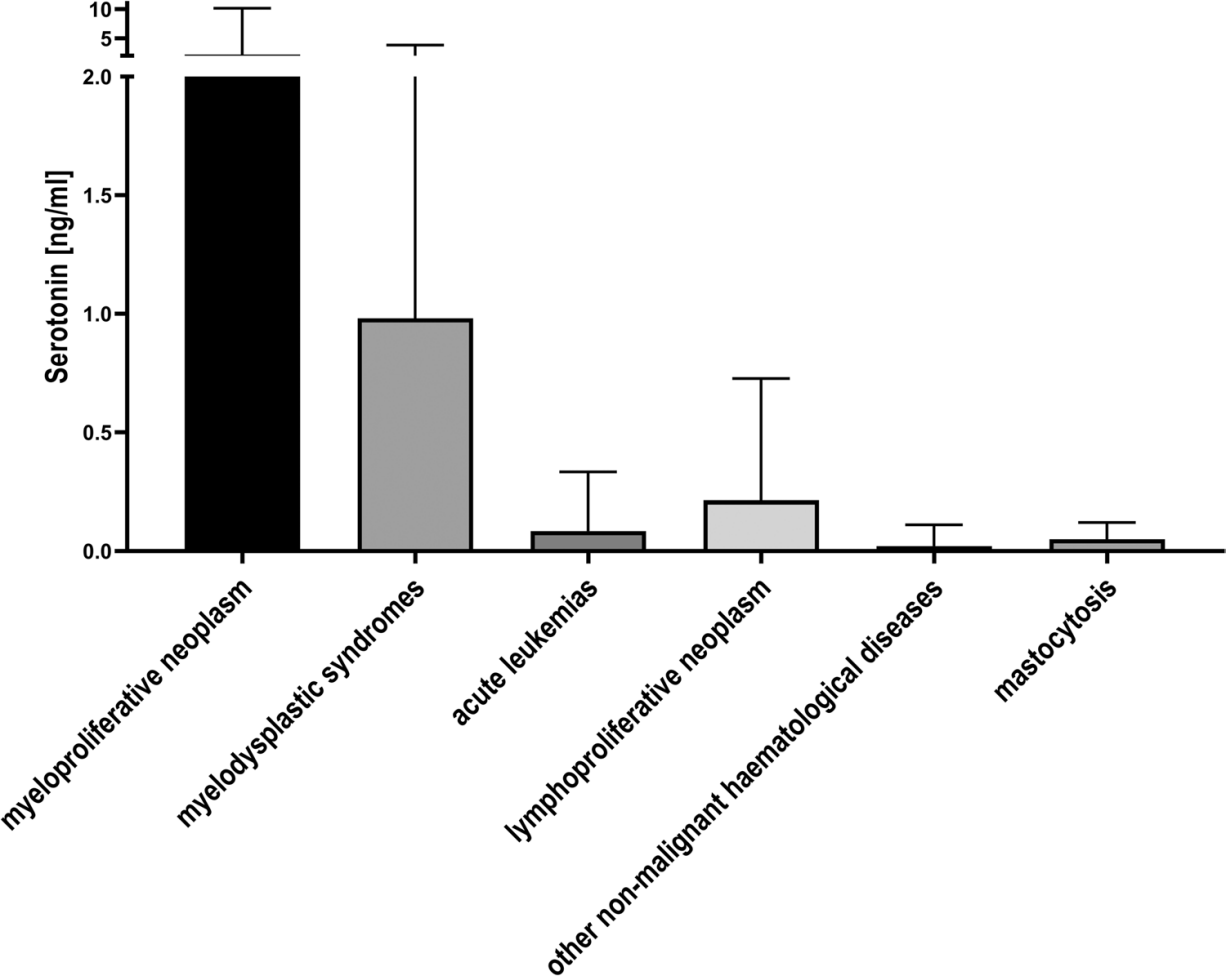
Box plot representing the correlation between serotonin levels and groups of hematological diseases

Statistical analysis showed that histamine levels were significantly higher in samples collected from patients with mastocytosis and myeloproliferative neoplasms than in samples from patients with myelodysplastic syndromes, acute leukemias, lymphoproliferative neoplasms, or other non-malignant hematological diseases (Fig. 3). However, no significant differences were found for serotonin levels between individual disease groups (Fig. 4).

### Correlation Between Histamine, Serotonin Concentration and Complete Blood Count Parameters

**Fig. 5:**
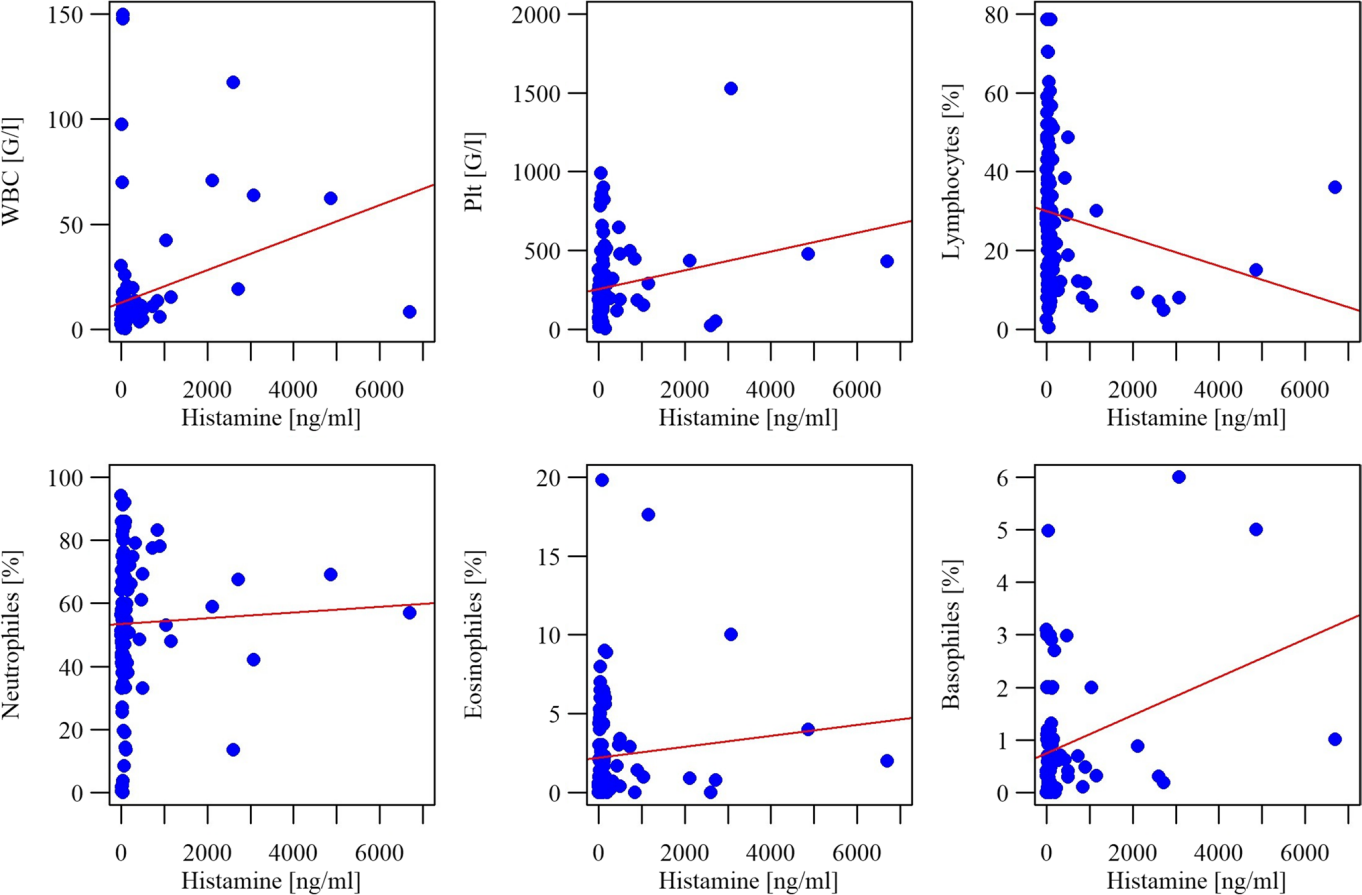
Correlations between histamine level and complete blood count parameters

**Fig. 6:**
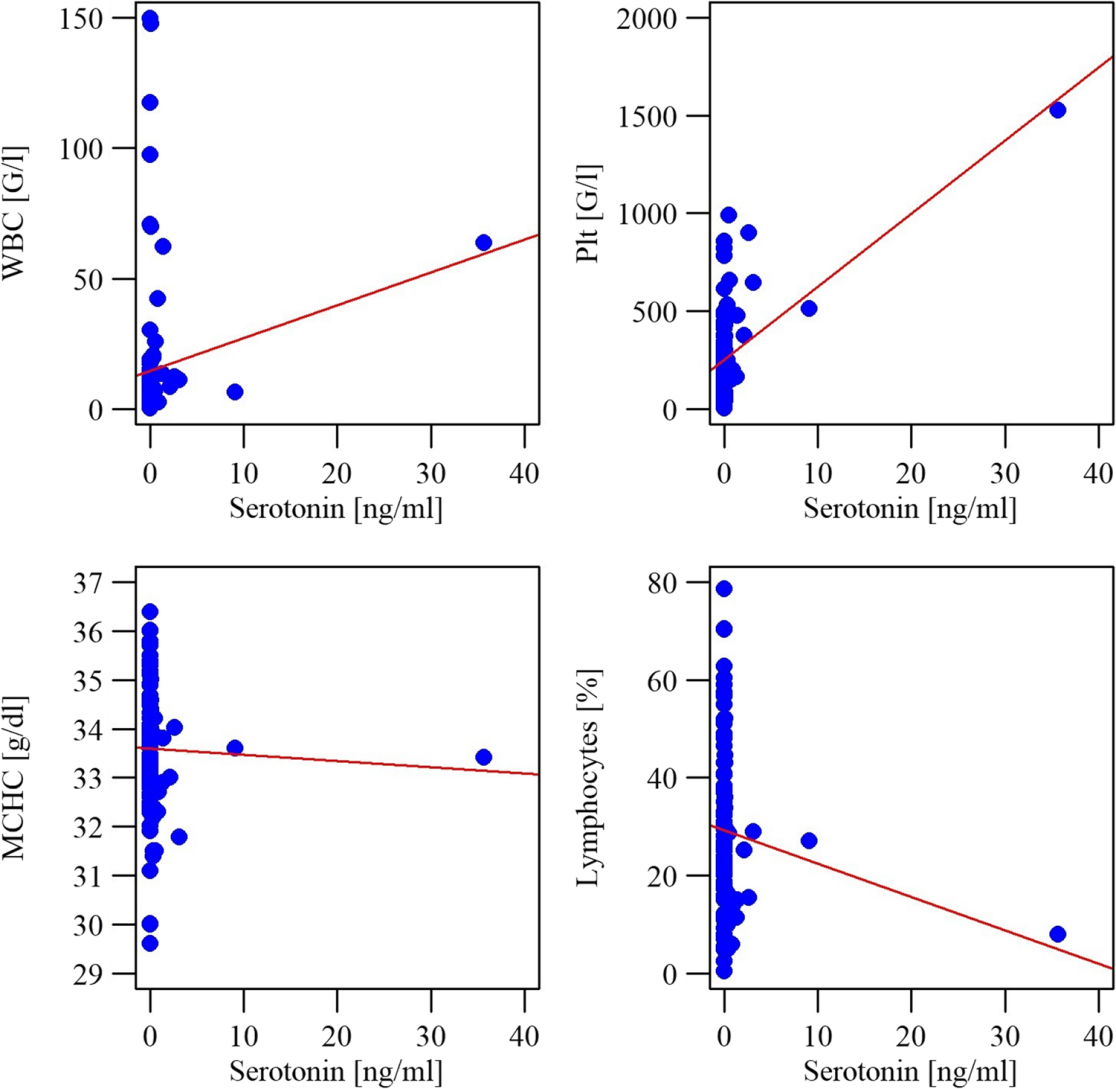
Correlations between serotonin level and complete blood count parameters

Statistical analysis showed a significant (p˂0.05) and positive (r˃0) correlation between the level of histamine and the white blood cell count, platelet count, as well as percentages of neutrophils, eosinophils and basophils. There was a significant (p˂0.05) and negative (r˂0) correlation between histamine levels and percentage of lymphocytes (Fig. 5). We found a significant (p˂0.05) and positive (r˃0) correlation between serotonin concentration and counts of white blood cells and platelets, and a significant (p˂0.05) negative (r˂0) correlation between serotonin and MCHC and the percentage of lymphocytes (Fig. 6).

## Discussion

Undoubtedly*,* the functions of cells should be closely associated with the substances produced, accumulated, released and/or transported by these cells. Findings from routine tests analyzing complete peripheral blood refer only to the counts and morphotic features of the cells under evaluation, which does not have to be synonymous with their physiological functionality. Only an additional evaluation of the levels of compounds specifically accumulated by these cells appears to precisely reflect their full potential for physiological activity. Although analytical techniques including flow cytometry allow for the determination of the degree of cell granularity, this method is unable to precisely define the chemical composition of cells. Dedicated diagnostic procedures (immunochemical or chromatographic methods) currently allow for the measurement of the concentration of specific chemical compounds produced by a given type of cells. It should be emphasized that the functionality of cells expressed by the level of specific compounds may refer not only to physiological conditions, but should also be related to pathogenic conditions. For example, in patients with cutaneous mastocytosis, itching, urticaria, and erythema result from the increased proliferation of mast cells in the skin and their degranulation followed by the release of histamine. Therefore, the measured levels of mediators may reflect not only their activity, but also the number of certain cells, indicating in this way a specific disease closely associated with a given cell type.

In this study we focused on two mediators, histamine and serotonin, and examined their concentrations in bone marrow niche for the first time. No data on reference levels for bone marrow were available at the moment of study, and therefore we used reference values for peripheral blood, which were determined with ELISA, LC–MS/MS, i.e. 20–200 ng/mL for histamine and 100–300 ng/mL for serotonin [9, 10]. In most analyzed cases, the levels of histamine measured in bone marrow were within the normal reference range determined for blood, which indicates that histamine is produced and accumulated already in the bone marrow. Our observations are consistent with findings by Hu Li et al. (2020), who reported that myeloid cells expressing histidine decarboxylase (HDC) are the dominant cells in bone marrow and maintain a high level of histamine in the bone marrow cavity [11]. On the other hand, serotonin levels measured in most bone marrow samples were below the limit of quantification of the method used (0.2 ng/mL), which is significantly lower than the reference values defined for blood. Thus, it is reasonable to claim that the synthesis of serotonin takes place in the extramedullary regions, and its potential levels in bone marrow higher than 0.2 ng/mL are unlikely to be a physiological feature. Our special attention was drawn to one case where the concentration of serotonin was much higher than in other samples (as much as 35.7 ng/mL), in addition to a high concentration of histamine (3083.7 ng/mL). This sample was collected from a patient diagnosed with chronic myeloid leukemia, specified as the megakaryocytic and granulocytic variant in the descriptive part of the myelogram. The high concentration of serotonin in this case could be associated with an abnormally high proliferation of megakaryocytes because these platelet precursors are able to synthesize serotonin *in vitro*, as reported by Kirouac et al. [12]. Although our study population included only one patient with this type of disease, findings appear to be very characteristic for this variant of disease and suggest that megakaryocytes can produce serotonin not only *in vitro,* but also *in vivo*.

Histamine and serotonin levels were correlated with specific groups of hematological diseases. Statistical analysis confirmed a significant correlation between the concentration of histamine in bone marrow niche and mastocytosis (Fig. 3). Mastocytosis is a disease characterized by an increased proliferation of mast cells, which are one of the main cells that produce histamine. In systemic mastocytosis, excessive accumulation of mast cells is observed in different organs, including bone marrow. Thus, the observed high histamine levels in bone marrow in patients with mastocytosis most likely result from the synthesis and accumulation of histamine in the granular components of mast cells already in the bone marrow, including cells with malignancy. Although the validity of our inference is limited because the number of cases in this group was small (n = 2) yet reflecting the low incidence of mastocytosis in the general population, it appears that histamine might be a useful biomarker in hematological diagnostics due to its high selectivity for systemic mastocytosis. Interestingly, patients with systemic mastocytosis had high levels of histamine only in bone marrow, with levels in complete blood/plasma showing no significant abnormalities.

We also found significant correlations between histamine levels in patients with myeloproliferative neoplasms, characterized by the proliferation of the myeloid lineage of hematopoietic stem/progenitor cells, including the precursors of histamine-synthesizing cells. It can be assumed that these cells produce histamine already in the bone marrow stem cell niche, and their increased proliferation leads to an increase in the concentration of histamine measured in bone marrow aspirates.

On the other hand, the statistical analysis of measured serotonin levels did not show significant correlations with the analyzed groups of hematological diseases (Fig. 4), although, as mentioned before, the increased level of serotonin could be associated with increased megakaryocytic proliferation.

Evaluation of histamine levels in relation to specific blood count parameters revealed a significant positive correlation with the counts of platelets, white blood cells, percentages of neutrophils, basophils, eosinophils, and a negative correlation with the percentage of lymphocytes (Fig. 5). It is known that neutrophils can synthesize histamine, but at concentrations much lower than those found in basophils and mast cells [13, 14]. In the physiological state, a portion of mature neutrophils (up to 2%) migrates to the bloodstream, while a significant pool of them remains in the bone marrow and is termed the bone marrow reserve [15]. The observed correlation indicates that histamine is produced by neutrophils still residing in bone marrow. As neutrophils account for the majority of white blood cells in the blood, this may explain the positive correlation with WBC. Similarly, basophils are among the main cells that synthesize and accumulate histamine and they can, in view of our findings, produce histamine in bone marrow. Both types of cells are released from bone marrow in a fully functional form, i.e. they contain adequate levels of histamine. Interestingly, a positive correlation of histamine levels was also found for eosinophils, regardless of the fact that they contain histaminase in the granular components. This observation appears to highlight the special role of individual cell lineages as storage sites for histamine. On the other hand, the main physiological effect of histamine is produced after cell degranulation and histamine release, and is not associated with the intracellular activity of histamine. The presence of both histamine and histaminase accumulated in eosinophils must be associated with a shorter activity of histamine after degranulation, compared to other granulocytes not containing histaminase.

Although some studies have reported a possible histamine production by lymphocytes [16, 17], our research does not provide evidence supporting the significant role of these cells in the accumulation and release of high amounts of histamine. On the contrary, a significant negative correlation was found between the concentration of histamine in bone marrow and the percentage of lymphocytes in the blood. This is most likely due to the fact that while lymphocytes can synthesize histamine *de novo*, they are not an accumulation site for histamine, unlike mast cells or basophils.

More detailed and complex research is required to explain the negative correlation between levels of serotonin in bone marrow and the percentage of lymphocytes and MCHC measured in peripheral blood. It should be noted that the MCHC in clinical terms is not interpreted alone, and in the studied group of patients the remaining red cell markers did not show any significant correlation with the concentration of serotonin in bone marrow.

However, statistical analysis revealed a positive, significant correlation between serotonin level and WBC, and the platelet count. It is known that platelets only transport serotonin produced outside bone marrow by enterochromaffin cells, but are unable to synthesize it [18, 19]. Therefore, our findings may confirm the results of studies on the *in vivo* synthesis of serotonin by its precursors located in bone marrow.

## Conclusion

Our study revealed for the first time that the concentration of histamine in bone marrow is comparable to the values measured in blood in a physiological state. We found very high levels of histamine only in patients with systemic mastocytosis, and linked it with an increased accumulation of mature mast cells in bone marrow. It is worth noting, that while histamine levels are typically in the range of 20–200 ng/mL, they are 20–30 times higher in patients with mastocytosis. Although the study group was small and further research is required for drawing final conclusions, such a huge disproportion of the measured levels and its obvious cause indicate the potential clinical value of histamine level as a selective and sensitive biomarker for systemic mastocytosis. We also noted high levels of histamine in patients with myeloproliferative diseases, which is consistent with the specific nature of these medical conditions and may result from the increased proliferation of histamine-containing cells. Serotonin, on the other hand, is not produced in significant amounts by bone marrow, and its increased levels may indicate an increased proliferation of megakaryocytes in bone marrow.

## Data Availability

Raw data is stored on local disks in the Forensic Medicine Department and is available on request. In this matter, please contact the corresponding author.
